# A Bayesian multivariate hierarchical model for developing a treatment benefit index using mixed types of outcomes

**DOI:** 10.1186/s12874-024-02333-z

**Published:** 2024-09-27

**Authors:** Danni Wu, Keith S. Goldfeld, Eva Petkova, Hyung G. Park

**Affiliations:** 1 Department of Population Health, New York University Grossman School of Medicine, 180 Madison Avenue, New York, 10016 New York USA; 2 Department of Biostatistics, Harvard T.H. Chan School of Public Health, 677 Huntington Avenue, Boston, 02115 MA USA

**Keywords:** Individualized treatment decision rule, Precision medicine, Treatment benefit index model, Bayesian multivariate hierarchical model, COVID-19

## Abstract

**Background:**

Precision medicine has led to the development of targeted treatment strategies tailored to individual patients based on their characteristics and disease manifestations. Although precision medicine often focuses on a single health outcome for individualized treatment decision rules (ITRs), relying only on a single outcome rather than all available outcomes information leads to suboptimal data usage when developing optimal ITRs.

**Methods:**

To address this limitation, we propose a Bayesian multivariate hierarchical model that leverages the wealth of correlated health outcomes collected in clinical trials. The approach jointly models mixed types of correlated outcomes, facilitating the “borrowing of information” across the multivariate outcomes, and results in a more accurate estimation of heterogeneous treatment effects compared to using single regression models for each outcome. We develop a treatment benefit index, which quantifies the relative benefit of the experimental treatment over the control treatment, based on the proposed multivariate outcome model.

**Results:**

We demonstrate the strengths of the proposed approach through extensive simulations and an application to an international Coronavirus Disease 2019 (COVID-19) treatment trial. Simulation results indicate that the proposed method reduces the occurrence of erroneous treatment decisions compared to a single regression model for a single health outcome. Additionally, the sensitivity analyses demonstrate the robustness of the model across various study scenarios. Application of the method to the COVID-19 trial exhibits improvements in estimating the individual-level treatment efficacy (indicated by narrower credible intervals for odds ratios) and optimal ITRs.

**Conclusion:**

The study jointly models mixed types of outcomes in the context of developing ITRs. By considering multiple health outcomes, the proposed approach can advance the development of more effective and reliable personalized treatment.

**Supplementary Information:**

The online version contains supplementary material available at 10.1186/s12874-024-02333-z.

## Introduction

In recent years, the growing emphasis on tailoring treatment strategies for patients according to their unique characteristics and disease manifestations has fueled a surge of interest among researchers and clinicians in the development of individualized treatment decision rules (ITRs) [1–12]. Methods for developing ITRs typically rely solely on a single health outcome, thus limiting the full exploitation of the available outcomes data, resulting in suboptimal data usage for individualized treatment decision-making.

To address this issue, we capitalize on the wealth of correlated and clustered health outcomes collected in trials by utilizing multivariate models. Multivariate models have demonstrated significant improvements in estimation and prediction accuracy compared to their univariate counterparts [13–19]. Although correlated and clustered observations are often modeled within the frequentist paradigm by a marginal model via generalized estimating equations or a generalized linear mixed model [20], Bayesian methods can handle highly complex hierarchical structures and efficiently estimate parameters via Markov Chain Monte Carlo sampling, making it an appealing strategy [21–23].

We propose a Bayesian multivariate hierarchical model for treatment effect heterogeneity to enable the “borrowing of information” among multiple correlated mixed types of outcomes, resulting in a more accurate estimation of treatment effects. Based on the proposed model, we employ a treatment benefit index [24, 25] to optimize ITRs.

Existing methods for ITRs in the presence of multiple outcomes have been proposed [26–36], including estimation of composite outcomes [34, 35], estimating patients’ outcome preferences [31, 33, 37], “set-valued” approaches [27, 28] and constrained estimation [26, 30] that focuses on balancing competing multiple outcomes. However, the emphasis of this paper is different in that we focus on improving the estimation efficiency through building the connection between correlated mixed types of outcomes using a Bayesian hierarchical model. This strategy is particularly effective when there is reason to believe that the treatment exerts similar influences on the outcomes. By accommodating dependency in multiple correlated health outcomes, our approach improves the estimation of treatment effects at both the patient and outcome-specific levels. Simulation results demonstrate the substantial gains in performance offered by the proposed hierarchical model. The method is applied to data from a clinical trial of COVID-19 convalescent plasma treatment. In the Continuous Monitoring of Pooled International Trials of Convalescent Plasma for COVID-19 Hospitalized Patients (COMPILE) trial [38–40], multiple correlated health outcomes were collected, including the primary ordinal outcome measure [41] and several secondary outcomes. By providing improved estimations of heterogeneous treatment effects and more accurately quantified uncertainty measurements that reflect all the available information from multiple health outcomes, the proposed modeling approach offers researchers a tool that allows taking advantage of the availability of multiple outcomes, in addition to patient characteristics, when optimizing treatment decisions for individual patients.

We organize the paper as follows. In “Methods” section, we present the Bayesian multivariate model for estimating heterogeneous treatment effects and developing ITRs and discuss the reasoning behind the selection of prior distributions. We also describe the simulation setup used to compare the performance of the proposed multivariate model with a univariate model for a single outcome, and sensitivity analyses to assess the robustness of the proposed model, as well as outlining an application to data from an international COVID-19 study, COMPILE. In “Results” section, we present extensive simulation results, including comparative analysis and sensitivity analyses, and the results from applying the proposed multivariate model to the COMPILE study, demonstrating its ability to provide more accurate estimations of heterogeneous treatment effects, as represented by odds ratios (ORs) with narrower credible intervals (CrIs) reflecting available correlated outcomes information. In “Discussion and conclusions” section, we provide a discussion on potential future applications of our work.

## Methods

In this section, we present a Bayesian approach for modeling mixed types of outcomes within the exponential family of distributions. Let $\varvec{Y}_i$ represent the vector of treatment outcomes of length *d* for the $i^{th}$ subject ($i =1,\ldots ,n$), where each element $Y_i^{(k)}$ ($k =1,\ldots ,d$) follows an exponential family distribution. Let $\varvec{\eta }_i = (\eta _i^{(1)},\ldots ,\eta _i^{(d)})^\top \in \mathbb {R}^d$, where $\eta _i^{(k)}$ is the canonical parameter associated with the assumed distribution of $Y_i^{(k)}$. Additionally, we define $\varvec{\phi } = (\phi ^{(1)},\ldots ,\phi ^{(d)})^\top \in \mathbb {R}^d$, where $\phi ^{(k)} > 0$ is an unknown dispersion parameter. We consider a vector of pre-treatment characteristics $\varvec{X}_i \in \mathbb {R}^p$ and the treatment indicator variable $A_i \in \{0,1\}$.

Conditional on $\varvec{\eta }_i$ and $\varvec{\phi }$, the *d* components of $\varvec{Y}_i = (Y_i^{(1)},\ldots ,Y_i^{(d)})^\top \in \mathbb {R}^d$ are assumed to be independent. The likelihood of $\varvec{y} = (\varvec{Y}_1^\top ,\ldots , \varvec{Y}_n^\top )^\top$ can be expressed as:

1 $$\begin{aligned} f(\varvec{y}|\varvec{\eta },\varvec{\phi }) & = \prod _{i=1}^n\prod _{k=1}^{d} f(Y_i^{(k)}|\eta _i^{(k)},\phi ^{(k)}) \nonumber \\ & = \prod _{i=1}^n\prod _{k=1}^{d}\exp \{[Y_i^{(k)}\eta _i^{(k)} - b_k(\eta _i^{(k)})]/a_k(\phi ^{(k)}) + c_k(Y_i^{(k)},\phi ^{(k)})\}, \end{aligned}$$

where $a_k(\cdot )$, $b_k(\cdot )$, and $c_k(\cdot )$ are the exponential family distribution-specific known functions for the $k^{th}$ outcome $Y_i^{(k)}$, whereas $\eta _i^{(k)} \in \mathbb {R}$ and $\phi ^{(k)} > 0$ are unknown quantities.

In Eq. (2), we relate the expected *k*th outcome with covariates $\varvec{X}_i$ and treatment assignment $A_i$, via a canonical parameter $\eta _i^{(k)}$ (defined below) and the corresponding canonical link function $g^{(k)}(\cdot )$ (e.g., identity function for a continuous outcome, logit function for a binary outcome, and log function for a count outcome):

2 $$\begin{aligned} \eta _i^{(k)} = g^{(k)}(\mathbb {E}[Y_i^{(k)}|\varvec{X}_i, A_i]) = \tau ^{(k)} + \varvec{X}_i^\top \varvec{m}^{(k)} + A_i(\beta _0^{(k)} +\varvec{X}_i^\top \varvec{\beta }^{(k)}). \end{aligned}$$

In model (2), $\tau ^{(k)} \in \mathbb {R}$ is the outcome-specific intercept, $\varvec{m}^{(k)} \in \mathbb {R}^p$ is the main effect of the pre-treatment characteristics $\varvec{X}_i$ on the $k^{th}$ outcome, $\beta _{0}^{(k)} \in \mathbb {R}$ is the main effect of the experimental treatment ($A = 1$) (vs. control $A = 0$) on the $k^{th}$ outcome, and $\varvec{\beta }^{(k)} \in \mathbb {R}^p$ is the *A*-by-$\varvec{X}$ interaction effect coefficient vector for the $k^{th}$ outcome.

For patients with pre-treatment characteristics $\varvec{x}$, the treatment-control effect contrast based on model (2) can be written as:

3 $$\begin{aligned} g^{(k)}(E(Y_i^{(k)}|\varvec{X}_{i} = \varvec{x},A_{i}=1)) - g^{(k)}(E(Y_i^{(k)}|\varvec{X}_{i} = \varvec{x},A_{i}=0)) = \beta _0^{(k)} + \varvec{x}^\top \varvec{\beta }^{(k)}. \end{aligned}$$

This treatment-control effect contrast is the primary focus in clinical trials. For example, if the outcome is binary and the function $g^{(k)}(.)$ is a *logit* link, $\beta _0^{(k)} + \varvec{x}^\top \varvec{\beta }^{(k)}$ corresponds to the effect of the experimental treatment (vs. control) on the $k^{th}$ outcome, as measured by the log odds ratio ($\log \textrm{OR}$). Without loss of generality, let us assume that the first outcome ($k=1$) is the primary outcome, in which a lower value of this outcome is preferable. Then, a $\log OR < 0$ signifies that the experimental treatment is expected to yield a more favorable primary outcome compared to the control treatment. Equation (3) indicates that the treatment-control effect contrast, e.g. $\log \textrm{OR}$, depends solely on the treatment *A*’s main effect ($\beta _0^{(k)}$) and the *A*-by-$\varvec{X}$ interactions ($\varvec{\beta }^{(k)}$), and does not depend on the $\varvec{X}$ main effects ($\varvec{m}^{(k)}$ in model (2)). The proposed Bayesian model’s objective is to efficiently estimate the effect of treatment *A* and the *A*-by-$\varvec{X}$ interaction effects on the primary outcome $Y^{(1)}$, by “borrowing information” from other correlated outcomes $Y^{(k')}, k' > 1$.

### Individualized treatment decision rule

Let us use $\mathcal {D}$ to denote the collection of the observed data from a clinical trial. Our goal is to predict optimal treatments for future patients, taking into account their pre-treatment characteristics. We define the treatment benefit index (TBI) for a patient with pre-treatment characteristics $\varvec{x}$ as the posterior probability that the treatment-control contrast in Eq. (3) is less than 0:

4 $$\begin{aligned} \text {TBI}(\varvec{x}) = Pr(\beta _0^{(1)} +\varvec{x}^\top \varvec{\beta }^{(1)} < 0 | \mathcal {D}), \end{aligned}$$

representing the posterior probability that the experimental treatment $(A=1)$ is more beneficial than the control treatment $(A=0)$. The estimated optimal ITR, denoted as $\hat{a}^{opt}: \varvec{x} \mapsto \{0,1\}$, is defined based on the TBI in Eq. (4):

5 $$\begin{aligned} \hat{a}^{opt}(\varvec{x}) = I(\text {TBI}(\varvec{x}) > \delta ), \end{aligned}$$

where *I*(.) is the indicator function, and $0< \delta <1$ is a threshold probability to make treatment decisions. We set the threshold $\delta$ to 0.5 in this paper. If the TBI exceeds 0.5, then the patient is recommended to receive the experimental treatment (i.e., $\hat{a}^{opt}(\varvec{x})=1$), as there is a more than 0.5 probability that the experimental treatment is more beneficial than the control treatment.

### Model and prior specification

In this section, we describe a framework for modeling mixed types of multivariate outcomes. The framework was motivated by the COMPILE study, in which we encountered the need to jointly model a primary ordinal outcome and binary outcomes. Although we demonstrate the applicability and utility of the proposed framework using ordinal and binary outcomes as an example, the framework is designed to be adaptable to other mixed outcome types.

To model the primary ordinal outcome with $L (=11)$ ordered levels of the study, a cumulative proportional odds model was determined to be the most appropriate method [42]. Let $Y^{(1)}$ represent the *L* levels ordinal outcome, with level-specific probabilities $P(Y_i^{(1)} = y) = p_{iy}^{(1)}$ for $y=0,\ldots ,L$. The cumulative probabilities are modeled as $\text {logit}(P(Y_i^{(1)} \ge y)) = \tau _y^{(1)} + \theta _i^{(1)}$, where $\tau _{y}^{(1)}$ $(y=1,\ldots ,L-1$) represent the level-specific intercepts subject to the monotonicity constraint of the cumulative logit model, and $\theta _i^{(1)}$ is a linear predictor defined below. Logistic models are used to analyze the binary outcomes. Let $Y^{(2)}, \ldots , Y^{(d)}$ denote the $d-1$ binary outcomes. Bernoulli distributions with probabilities $P(Y_i^{(k)} = 1) = p_i^{(k)}$ $(k=2,\ldots , d)$ are used, modeled as $\text {logit}(p_i^{(k)}) = \tau ^{(k)} + \theta _i^{(k)}$, in which $\tau ^{(k)}$ are the intercepts and $\theta _i^{(k)}$ $(k=2,\ldots ,d)$ are the linear predictors defined below.

6 $$\begin{aligned} Y_i^{(1)} & \sim \text {Ordinal multinomial}(\varvec{p}_i), \quad \varvec{p}_i =\{p_{iy}^{(1)}\}_{y=0}^{L-1} \nonumber \\ Y_i^{(k)} & \sim \text {Bernoulli}(p_i^{(k)}), \quad k = 2,\ldots , d \nonumber \\ logit(P(Y_i^{(1)} \ge y)) & =\tau ^{(1)}_y + \theta _i^{(1)}, \quad y=1,\ldots ,L-1 \nonumber \\ logit(P(Y_i^{(k)}=1)) & = \tau ^{(k)}+\theta _i^{(k)}, \quad k = 2,\ldots , d \nonumber \\ \theta _i^{(k)} & = \varvec{X}_i^\top \varvec{m}^{(k)}+ A_i\beta _{0}^{(k)} +A_i\varvec{X}_i ^\top \varvec{\beta }^{(k)}, \quad k=1,\ldots , d \nonumber \\ \varvec{m}^{(k)} & \sim \text {MVN}(\varvec{\mu }=\textbf{0},\Sigma =\ 2.5^2 I_{p\times p}) \nonumber \\ ( \beta _{j}^{(1)},\, \ldots ,\, \beta _{j}^{(d)} )^\top & \sim \text {MVN} ( \varvec{\mu }= \beta _j^{*}\textbf{1}_d, \Sigma = \sigma _{\beta _j}^2I_{d \times d} ), \quad j = 0,\ldots ,p \nonumber \\ \sigma _{\beta _j} & \sim \text {exponential}(\mu = 1) \nonumber \\ \beta _j^{*} & \sim \text {Normal}(\mu =0,\sigma =2.5) \nonumber \\ \tau _y^{(1)} & \sim t_{\text {student}}(df=3,\mu =0,\sigma =8), \quad y=1,\ldots ,L-1 \nonumber \\ \tau ^{(k)} & \sim t_{\text {student}}(df=3,\mu =0,\sigma =8), \quad k = 2,\ldots , d. \end{aligned}$$

**Outcome-specific treatment main effect** $\beta _0^{(k)}$ **and interaction effect** $\varvec{\beta }^{(k)}$: To facilitate flexible information sharing of the coefficients across outcomes, we employ hierarchical shrinkage. The prior distribution assumes that each outcome-specific treatment main effect $\beta _{0}^{(k)}$ is centered around a pooled “treatment main effect” $\beta _{0}^{*}$. The variation of each outcome-specific treatment main effect around the mean $\beta _{0}^{*}$ is represented by its standard deviation $\sigma _{\beta _{0}}$. The outcome-specific interaction effect $\beta _j^{(k)}$ ($j = 1, \ldots ,p$) is distributed as $\text {Normal}(\mu =\beta _{j}^{*}, \sigma =\sigma _{\beta _{j}})$, where $\beta _{j}^{*}$ denotes the pooled “interaction effect” across all *d* outcomes, and $\sigma _{\beta _j}$ controls the strength of information borrowing across the *d* outcomes. A large prior mean for $\sigma _{\beta _j}$ allows for greater variability, whereas a small value constrains the coefficients to remain closer to the pooled effect. In “ Simulation” section, we assigned a prior mean of 1 to $\sigma _{\beta _j}$.

For the outcome-specific intercepts $\tau ^{(k)}$, we use a $t_{\text {student}}$ distribution with 3 degrees of freedom ($\sigma = 8$). This choice offers heavier tails compared to the *Normal* distribution ($\sigma = 8$), ensuring the Hamiltonian Monte Carlo (HMC) sampling [43] to have adequate flexibility for exploring the sample space. In the case of covariates’ main effects $\varvec{m}^{(k)}$, we use a diffuse prior, with the expectation that the observed data will primarily determine the posterior distribution. Similarly, for the pooled treatment main effect and interaction effects across outcomes ($\beta _j^{*}$), we adopt a diffuse prior. The Bayesian models were implemented using Stan [43], which enables Bayesian inference based on HMC, with the No-U-Turn sampler [43].

### Simulation

In this section, we present a comparative analysis of two Bayesian models for estimating heterogeneous treatment effects and ITRs. Specifically, we compared the performance of the proposed multivariate model to that of a univariate model, which only relies on a single primary outcome. We also conducted sensitivity analyses to assess the robustness of the proposed model across different study scenarios.

#### Simulation setup and performance evaluation

We used the R package *simstudy* [44] to generate simulated data sets. For a given training sample size *n*, we independently generated treatment indicators, denoted $A_i \in \{0,1\}$, from the Bernoulli distribution with a probability of $P(A_i=1) = 0.5$. The covariates $\varvec{X}_i \in \mathbb {R}^p$ comprised 3 independent binary variables generated from the Bernoulli distribution with probability $P(X_i=1) = 0.5$, and $p-3$ independent continuous variables, drawn from the multivariate normal distribution with mean zero and unit variance. We consider $p=5$ covariates. We generated a set of four outcomes $(Y_i^{(1)}, Y_i^{(2)}, Y_i^{(3)}, Y_i^{(4)})$, mimicking the outcomes collected from the COMPILE study. The variable $Y_i^{(1)}$ follows an 11-level ordinal multinomial distribution, while $Y_i^{(2)}$, $Y_i^{(3)}$, and $Y_i^{(4)}$, representing the 3 supplementary binary outcomes, are generated using Bernoulli distributions. The true parameter values used, with the notations adhering to model (6), for the data generation are as follow. The covariates’ main effect coefficients for each of the 4 outcomes are $\varvec{m}^{(1)} = [ 0.35, -0.40, 0.15, 0.20, -0.21 ]^\top$, $\varvec{m}^{(2)} = [ 0.40, -0.38, 0.13, 0.19, -0.22 ]^\top$, $\varvec{m}^{(3)} = [ 0.38, -0.39, 0.14, 0.18, -0.20 ]^\top$, $\varvec{m}^{(4)} = [ 0.42, -0.41, 0.16, 0.21, -0.19 ]^\top$.

- Treatment’s main effect coefficient for each (the *k*th) outcome:
  - $$\beta _0^{(1)} = -0.05$$
  - $$\beta _0^{(2)} = -0.06$$
  - $$\beta _0^{(3)} = -0.03$$
  - $$\beta _0^{(4)} = -0.04$$
- *A*-by-$\varvec{X}$ interaction effect coefficients for each (the *k*th) outcome:
  - $$\varvec{\beta }^{(1)} = \left[ \begin{array}{lllll} 0.20&-0.10&0.10&0.05&-0.06 \end{array}\right] ^\top$$
  - $$\varvec{\beta }^{(2)} = \left[ \begin{array}{lllll} 0.19&-0.11&0.09&0.04&-0.07 \end{array}\right] ^\top$$
  - $$\varvec{\beta }^{(3)} = \left[ \begin{array}{lllll} 0.18&-0.12&0.11&0.06&-0.05 \end{array}\right] ^\top$$
  - $$\varvec{\beta }^{(4)} = \left[ \begin{array}{lllll} 0.21&-0.09&0.12&0.07&-0.04 \end{array}\right] ^\top$$

As a comparison model for model (6), we employed a Bayesian univariate model (7) that only uses the single primary ordinal outcome, specified as follows:

7 $$\begin{aligned} Y_i^{(1)} & \sim \text {Ordinal multinomial}(\varvec{p}_i), \quad \varvec{p}_i =\{p_{iy}^{(1)}\}_{y=0}^{L-1} \nonumber \\ logit(P(Y_i^{(1)} \ge y)) & =\tau ^{(1)}_y + \theta _i^{(1)} \nonumber \\ \theta _i^{(1)} & = \varvec{X}_i^\top \varvec{m}^{(1)}+ A_i\beta _{0}^{(1)} +A_i\varvec{X}_i ^\top \varvec{\beta }^{(1)} \nonumber \\ \varvec{m}^{(1)} & \sim \text {MVN} ( \varvec{\mu }= \varvec{0}, \Sigma = 2.5^2I_{p \times p} ) \nonumber \\ \beta _0 & \sim \text {Normal}(\mu =0,\sigma =2.5) \nonumber \\ \varvec{\beta }^{(1)} & \sim \text {MVN}(\mu =\varvec{0}, \Sigma = 2.5^2I_{p\times p}) \nonumber \\ \tau _y^{(1)} & \sim t_{\text {student}}(df=3,\mu =0,\sigma =8). \end{aligned}$$

As evaluation metrics for the performance of the models, we considered two criteria: 1) the proportion of correct decisions (PCD); and 2) the area under the receiver operating characteristic (ROC) curve (AUC). The PCD corresponds to the proportion of cases with $\hat{a}^{opt}(\varvec{x}_i) =a^{opt}(\varvec{x}_i)$. Here, the true optimal ITR is defined as ${a}^{opt}(\varvec{x}_i) =I(\text {OR}(\varvec{x}_i) <1)$, in which $\text {OR}(\varvec{x}_i) =\exp (\beta _0^{(1)}+\varvec{x}_i^\top \varvec{\beta }^{(1)})$ where $\beta _0^{(1)}$ and $\varvec{\beta }^{(1)}$ correspond to the true values used in the data generation process, and the estimated ITR $\hat{a}^{opt}(\varvec{x}_i)$ is specified in Eq. (5) with the threshold $\delta =0.5$. Since we assumed (without loss of generality) a lower value of the outcome is desirable, an $\text {OR}(\varvec{x}_i) <1$ indicates that the experimental treatment is expected to yield a more desirable outcome than the control treatment for subject *i*.

PCD is computed using a decision threshold $\delta = 0.5$ as per Eq. (5). Another evaluation metric is the area under the curve (AUC), which does not rely on the selection of a specific decision threshold and accounts for the trade-off between true positive rate (sensitivity) and false positive rate (1 - specificity) for various decision thresholds. AUC values range from 0 to 1, with a higher value indicating a better classification performance [45]. To calculate the AUC, we first train the TBI as defined in Eq. (4), and then evaluate the TBI on the test data and generate the ROC curve, considering every unique TBI value as a potential threshold; for each threshold, we compute $\hat{a}^{opt}(\varvec{x}$) according to Eq. (5), and compare it with $a^{opt}(\varvec{x}$) to calculate the true positive and false positive rates. Then the *auc* function from the *pROC* package [46] is used to compute the AUC.

We conducted simulation for various training sample sizes, $n \in \{250, 500, 1000, 2000\}$, and a fixed test dataset size of 2000. For each *n*, we conducted 1000 simulations, with each simulation using 2000 HMC iterations for warm-up and retaining 10000 iterations for inference (all simulations in this paper used the same number of HMC iterations). The Stan code for the Bayesian multivariate hierarchical model is provided in Additional file 1.

#### Sensitivity analyses

Each patient’s individual-level treatment efficacy for a specific outcome can vary, making it logical to incorporate random effects into the data generation process. In this section, we conducted sensitivity analyses to assess the robustness of the models. To simulate various study scenarios, we introduce a modified data generation model that incorporates additional parameters, $\gamma _{i0}$ and $\varvec{\Gamma }_i$:

8 $$\begin{aligned} \theta _i^{(k)} = \varvec{X}_i^\top \varvec{m}^{(k)} + A_i(\beta _0^{(k)}+\gamma _{i0}) +A_i\varvec{X}_i ^\top (\beta ^{(k)} +\varvec{\Gamma }_i) \end{aligned}$$

The $\gamma _{i0}$ indicates the random effect associated with treatment, and $\varvec{\Gamma }_i$ indicates the random effect associated with *A*-by-$\varvec{X}$ interaction. The element-wise standard deviation for both random effects is determined by $\sigma$. The true values of the other parameters follow the data generation process described in “Simulation setup and performance evaluation” section. We considered a range of values for $\sigma \in \{0.1, 0.2, 0.3\}$, as well as different training sample sizes $n \in \{250, 500, 1000, 2000\}$, with a fixed test dataset size of 2000. For each set of $\sigma$ and *n*, we conducted 1000 simulations. The PCD and AUC were used for model performance evaluation.

### Application to data from a COVID-19 randomized clinical trial

In this section, we apply the proposed Bayesian multivariate model to data from $n=2341$ patients in the COMPILE COVID-19 clinical trial, focusing on the COVID-19 convalescent plasma (CCP) treatment for hospitalized COVID-19 patients not on mechanical ventilation at the time of randomization [38–40]. This study collected several mixed types of outcomes, including a primary outcome and supplementary/secondary outcomes. Park et al. [24] developed an ITR solely based on the primary ordinal outcome using a frequentist method. The current paper also focuses on the primary outcome. However, the proposed approach reduces the uncertainty associated with the estimation of heterogeneous treatment effects and ITRs by jointly modeling the mixed types of outcomes using Bayesian techniques and “borrowing information” across correlated outcomes.

The primary outcome is the World Health Organization (WHO) 11-point clinical scale, measured at 14 ± 1 day after randomization (hereafter, day 14), assessing COVID-19 severity with values ranging from 0 (no infection) to 10 (death) [47]. To “borrow information” we employ binary outcomes collected in the COMPILE study, such as hospitalization, ventilation or worse, and death at 28 ± 2 days after randomization (hereafter, day 28). We used the same set of pre-treatment characteristics as in the ITR from Park et al. [24], which was selected via extensive cross-validation. The pre-treatment characteristics are listed below.

- Pre-treatment characteristics in the treatment-by-$\varvec{X}$ interaction effects term: WHO score at baseline (an ordinal variable represents hospitalized but no oxygen therapy required, hospitalized with oxygen required via mask or nasal prongs, and hospitalized with high-flow oxygen required); WHO score at baseline & Age $\ge$ 67 interaction; Indicator for blood type A or AB; Indicator for the presence of cardiovascular disease; Indicator for comorbid diabetes mellitus & pulmonary disease.
- Pre-treatment characteristics in the main effects term: Age (mean (standard deviation) of 60.3 (15.2) years); Sex (35.7% were women); WHO score at baseline; WHO score at baseline & Age interaction; Indicator for blood type A or AB; Indicator for comorbid diabetes mellitus & cardiovascular disease interaction; Indicator for comorbid diabetes mellitus & pulmonary disease interaction; Duration of symptoms before randomization (a binary variable defined as 0-6 days and $\ge$ 7 days); Quarter during which patient was enrolled (a categorical variable that represents Jan-March 2020, Apr-June 2020, July-Sept 2020, Oct-Dec 2020, and Jan-March 2021); Indicator of treatment (a binary variable with 1 for CCP treatment, and 0 for control treatment).

We evaluated the performance of the two models: the multivariate model (6) and the univariate model (7). As it is expected that the main effect of treatment ($\beta _0^{(k)}$) should not vary significantly across different outcomes and interaction effects ($\beta _j^{(k)}$) exhibit relatively small variation across outcomes, we employed an informative prior $\sigma _{\beta _j} \sim \text {exponential} (\mu =0.3)$ on the hierarchically defined standard deviation parameter $\sigma _{\beta _j}$.

We assessed the goodness-of-fit for both the Bayesian multivariate and univariate models, Models (6) and (7), using posterior predictive checking, a method that evaluates the model’s ability to generate replicated data that closely resembles the observed data [40, 48–50].

## Results

In this section, we present simulation results of two Bayesian models for estimating heterogeneous treatment effects and ITRs. We also present sensitivity analyses results for evaluating the robustness of the proposed model across different study scenarios. We then applied the proposed model to an international COVID-19 clinical trial and examined the goodness-of-fit.

### Simulation results

**Fig. 1:**
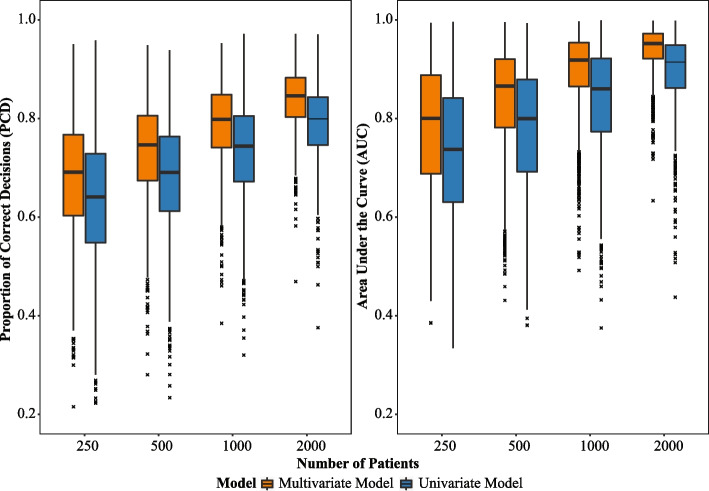
Boxplots of the proportion of correct decisions (PCD) and area under the curve (AUC) in the test sets, comparing the multivariate (orange) and univariate (blue) models across different training set sizes (as indicated in the x-axis). Each box shows the interquartile range (IQR), with the horizontal line inside the box representing the median PCD and AUC value. The whiskers extend to the minimum and maximum PCD and AUC values within 1.5 times the IQR. Outliers are represented by small cross symbols

The plot in Fig. 1 presents a comparison of the performance of the multivariate model (6) and the univariate model (7) based on their PCD and AUC values in the test sets. The simulation setup was described in “Simulation setup and performance evaluation” section. The performance is evaluated across varying training set sizes, represented by the number of subjects in the training set on the x-axis. The y-axis displays the PCD or AUC values, with higher values indicating better model performance. The figure illustrates that the multivariate model (in orange) generally exhibits higher PCD and AUC values compared to the univariate model (in blue) across all training set sizes, suggesting that the proposed multivariate model outperforms its univariate counterpart with respect to making correct treatment decisions for subjects in the test set.

In addition to achieving higher PCD and AUC compared to the univariate model (7), the proposed multivariate model (6) also reduces the uncertainty associated with the estimation of treatment effects. To evaluate this, we conducted 1000 simulations in each study scenario and computed the following metrics: (a) the average length of the 95% credible intervals (CrIs) for $\beta _0^{(1)}$ across simulations; (b) the coverage rate, which is the percentage of simulations where the true value of the treatment effect ($\beta _0^{(1)}$) falls within the estimated 95% CrIs; and (c) the mean squared error (MSE) between the estimated posterior median of the treatment effect and the true value. The simulation results, summarized in Additional file 2, indicate that the proposed multivariate approach provides narrower credible intervals and lower MSE, while maintaining high coverage rate greater than 95%.

Some experts believe that the true optimal ITR should be based on potential outcomes. In light of this perspective, we also provide a comparison of the performance of the Bayesian multivariate and univariate models utilizing the new potential outcomes-based ITR in Additional file 3. Despite the less remarkable improvement in PCD and AUC, the proposed model (6) still outperforms the univariate model (7).

#### Sensitivity analyses results

**Fig. 2:**
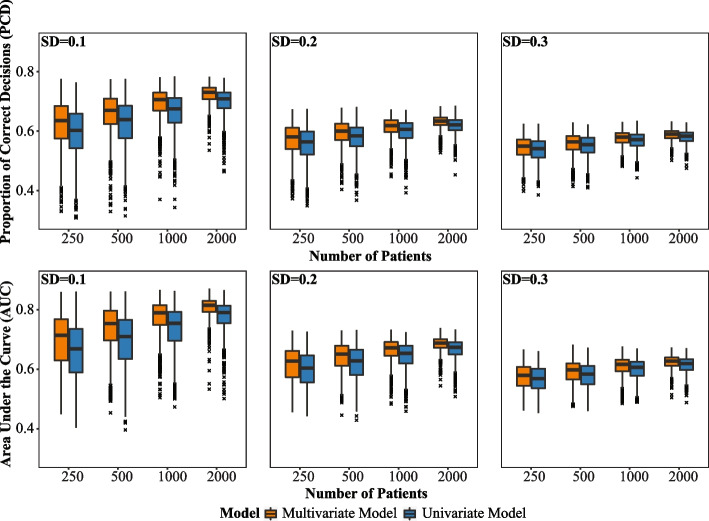
Boxplots of proportion of correct decisions (PCD) and area under the curve (AUC) in the test sets, comparing the multivariate (orange) and univariate (blue) models across different training set sizes (as indicated in the x-axis) and different standard deviations (SDs) of random effects. Three different levels of SD for random effects are considered in the data generation process: SD=0.1, SD=0.2, and SD=0.3

The PCD and AUC for sensitivity analyses, detailed in “Sensitivity analyses” section, are presented in Fig. 2. In the plot, the y-axis represents PCD or AUC, while the x-axis displays the number of subjects in the training set. The multivariate model (6) consistently outperforms the univariate model (7). However, when $\sigma$ = 0.2 and 0.3, the superiority of the multivariate model becomes less pronounced. This is because the true values of the main effect of treatment and fixed effects of the interaction terms are all $\le$ 0.21, and $\sigma$ = 0.2 and 0.3 already constitute relatively large values of random individual effects. Even with such a relatively large $\sigma$ value, the proposed model (6) still outperforms the univariate model (7), demonstrating the robustness of our approach. Using the same setting of sensitivity analyses, we also provide a comparison of the performance of multivariate model (6) and univariate model (7) utilizing the potential outcomes-based ITR in Additional file 4.

It is crucial to identify prior distribution assumptions. We conducted extensive simulations under different study composition scenarios to select prior distributions. We settled on a final set of prior distributions that consistently provided the highest PCD, AUC, and the fewest divergent transitions [51, 52] across a range of study scenarios. Given the extensive range of prior distributions we tested, we used one different set of prior distributions as an example to illustrate the robustness of our model’s results. This analysis can be found in Additional file 5.

### Application results for a COVID-19 randomized clinical trial

Our analysis used complete cases, yielding a final sample of 2287 patients (the number of patients at different clinical stages of COVID-19 measured on the WHO 11-point scale at day 14 by treatment group is provided in Additional file 6).

**Fig. 3:**
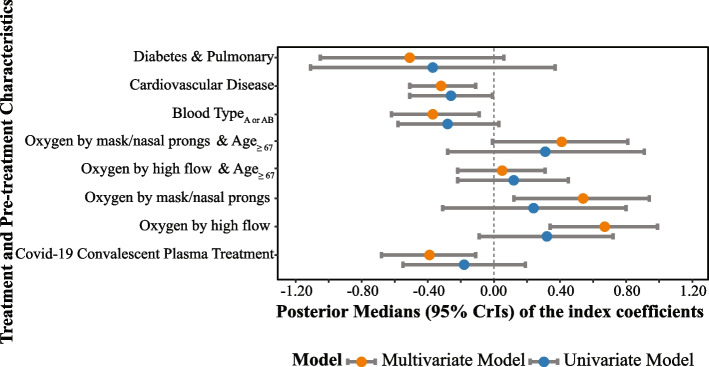
Comparison of univariate and multivariate models with respect to posterior distributions of coefficients that consistute the treatment benefit index (TBI), summarized by the posterior medians and 95% credible intervals (CrIs)

In Fig. 3, we presented the posterior distributions (medians and 95% CrIs) of coefficients ($\beta _0^{(1)}$ and $\varvec{\beta }^{(1)}$) for treatment and pre-treatment patient characteristics associated with the TBI for the primary ordinal outcome from both models, (6) and (7). Table 1 presents the posterior distributions of coefficients for treatment and pre-treatment characteristics for all ordinal and binary outcomes.

Figure 3 indicates that the multivariate model offers better precision when estimating coefficients for *A*-by-$\varvec{X}$interaction effect and treatment’s main effect in comparison to the univariate model, as reflected in narrower 95% CrIs, and most of the coefficients’ 95% CrIs do not include zero, unlike those of the univariate model. In contrast, for the univariate model, the 95% CrIs for almost all coefficients include zero. If the 95% CrI for the main treatment effect coefficient includes zero, we cannot draw a definitive conclusion about whether patients with the reference level of pre-treatment characteristics benefit more from CCP than from the control treatment. Similarly, if the 95% CrI for an *A*-by-*X* interaction effect coefficient includes zero, we cannot conclude whether patients with this specific pre-treatment characteristic benefit more from CCP than those without such pre-treatment characteristic.

As shown in Table 1, the estimated index coefficient for CCP treatment using the proposed multivariate model is $-0.39$. For patients with the reference level of pre-treatment characteristics, the CCP treatment effect can be measured by an OR with a posterior median of $\exp (-0.39)= 0.68 < 1$. This OR less than 1 indicates that the CCP treatment decreases the odds of experiencing a worse outcome compared to the control treatment for these patients, when the patient characteristics are set at their reference levels. For patients who have cardiovascular disease, in addition to the reference levels of the other pre-treatment characteristics, the CCP treatment effect is more effective. Specifically, in the presence of cardiovascular disease, CCP reduces the odds of a worse outcome by a multiplicative factor of $\exp (-0.32) = 0.73$, corresponding to an additional 27% reduction in the odds of a worse outcome when treated with CCP compared to patients without cardiovascular disease. The findings from the multivariate model are consistent with the results reported by Park et al. [24]: patients with pre-existing conditions, such as cardiovascular disease (the posterior median of the multiplicative change in treatment effect $\text {OR}= \exp (-0.32)= 0.73 < 1$), diabetes & pulmonary ($\exp (-0.51)=0.60 < 1$), blood type A or AB ($\exp (-0.37)=0.69 < 1$), and those at an early stage of COVID-19, represented by the indicator of hospitalized but no oxygen therapy required, are expected to benefit from CCP treatment significantly more than those without the specified pre-existing conditions and/or at later stage of COVID-19 ($\exp (0.54)=1.72 > 1$ and $\exp (0.67)=1.95 > 1$). In addition, the proposed Bayesian model provides lower levels of uncertainty in the estimation of the ORs.

**Fig. 4:**
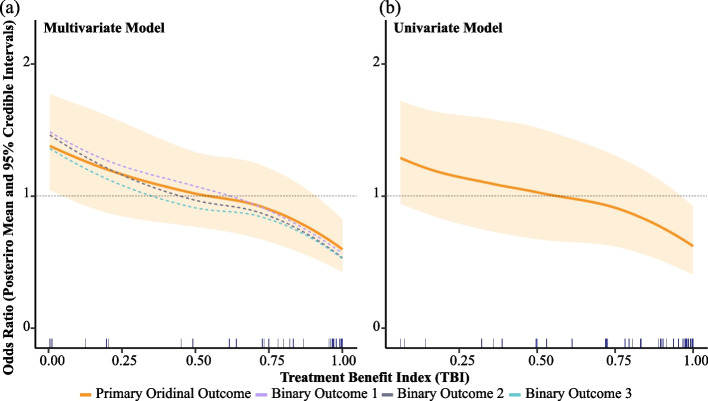
Posterior distributions of the treatment effect Odds Ratios (ORs) as a function of the treatment benefit index (TBI) derived from the multivariate model (left plot) and univariate model (right plot). In each plot, the solid curve represents the posterior mean of OR for the primary ordinal outcome, and the colored band represents the 95% credible interval (CrI) of this OR curve. The dashed curves in (a) correspond to the posterior means of the ORs for three supplementary binary outcomes. These supplementary outcomes are as follow: (1) the binary outcome of hospitalization at day 28, (2) the binary outcome of ventilation or worse at day 28, and (3) the binary outcome of mortality at day 28. The locally weighted smoothing (loess) method is applied to illustrate the overall trends. Rug plots at the bottom of each plot represent the data density along the x-axis. An OR for the active treatment (CCP) efficacy below 1 (dashed grey horizontal line) indicates a more favorable outcome with CCP treatment compared to the control treatment

For each patient, the effect of CCP treatment versus control on each outcome, as measured by OR, is calculated based on the patient’s pre-treatment characteristics and the posterior distributions of coefficients derived from either the multivariate or the univariate model. The TBI is subsequently computed in accordance with Eq. (4). Figure 4 presents a side-by-side comparison of the fitted models, illustrating the relationship between the TBI and the posterior mean of the OR for different outcome types in COMPILE. The left plot is based on the proposed multivariate model (6), in which the x-axis represents the TBI. The right plot is based on the univariate model (7). An odds ratio for CCP efficacy below 1 (dashed grey horizontal lines) indicates a more favorable outcome with CCP treatment compared to the control treatment, and the degree of treatment benefit from CCP is monotonically parameterized by the TBI.

A notable observation from Fig. 4 is the narrower 95% credible interval of the OR for the primary ordinal outcome when employing the multivariate model (6), compared to the univariate model (7). This suggests that the multivariate model incorporates and reflects richer available information from the multiple outcomes collected in the trial. Consequently, this improved accuracy may contribute to more informed clinical decision-making based on a more reliable representation of the relationship between CCP efficacy and TBI.

We assessed the goodness-of-fit for both the Bayesian multivariate and univariate models, Models (6) and (7), using posterior predictive checking, a method that evaluates the model’s ability to generate replicated data that closely resembles the observed data [40, 48–50]. The Bayesian p-value was employed to measure the model’s fit, with values near 0.5 suggesting a satisfactory fit. A detailed explanation of the procedure and the results of posterior predictive checking for both the Bayesian multivariate and univariate models is provided in Additional file 7. The results show that both models fit the data well.

**Table 1:** The estimated treatment benefit index (TBI) coefficients (Posterior Median [95% credible intervals]) for treatment and pre-treatment characteristics, under univariate and multivariate models (for all 4 outcomes). A positive coefficient suggests that the variable associated with this coefficient increases the odds of a higher category (i.e., worse) outcome. Conversely, a negative coefficient indicates that variable linked with this coefficient decrease the odds of a higher category (i.e., worse) outcome. The coefficient for COVID-19 convalescent plasma (CCP) treatment is negative, it indicates that patients with the reference level of pre-treatment conditions benefit more from CCP than from the control treatment. When the coefficient for a specific pre-treatment condition is negative, it means that (adjusting for the impact of the other conditions) patients with this specific pre-treatment condition benefit more from CCP in comparison to the CCP benefit for those without this pre-treatment condition

Treatment and pre-treatment characteristics	Index coefficients (Posterior Median [95%CrI])				
	Univariate model	Multivariate model			
	Primary ordinal outcome	Primary ordinal outcome	Binary outcome 1	Binary outcome 2	Binary outcome 3
Diabetes & Pulmonary	-0.37 [-1.11, 0.37]	-0.51 [-1.05, 0.06]	-0.51 [-1.10, 0.18]	-0.61 [-1.37, 0.00]	-0.65 [-1.56, -0.06]
Cardiovascular Disease	-0.26 [-0.51, -0.01]	-0.32 [-0.51, -0.11]	-0.35 [-0.59, -0.11]	-0.37 [-0.67, -0.15]	-0.37 [-0.68, -0.14]
Blood Type $_{\text {A or AB }}$	-0.28 [-0.58, 0.03]	-0.37 [-0.62, -0.09]	-0.47 [-0.82, -0.19]	-0.49 [-0.89, -0.20]	-0.43 [-0.78, -0.12]
Oxygen by high flow^a^ & Age$_{\ge \text {67 }}$	0.31 [-0.28, 0.91]	0.41 [-0.01, 0.81]	0.46 [0.03, 0.93]	0.46 [0.03, 0.92]	0.43 [-0.05, 0.87]
Oxygen by mask or nasal prongs^a^ & Age$_{\ge \text {67 }}$	0.12 [-0.22, 0.45]	0.05 [-0.22, 0.31]	0.06 [-0.24, 0.40]	-0.02 [-0.42, 0.28]	-0.01 [-0.42, 0.29]
Oxygen by high flow^a^	0.24 [-0.31, 0.80]	0.54 [0.12, 0.94]	0.56 [0.12, 1.00]	0.59 [0.15, 1.04]	0.56 [0.11, 1.01]
Oxygen by mask or nasal prongs^a^	0.32 [-0.09, 0.72]	0.67 [0.34, 0.99]	0.75 [0.40, 1.16]	0.75 [0.37, 1.16]	0.71 [0.32, 1.11]
COVID-19 Convalescent Plasma (CCP) Treatment	-0.18 [-0.55, 0.19]	-0.39 [-0.68, -0.11]	-0.41 [-0.73, -0.10]	-0.41 [-0.74, -0.10]	-0.44 [-0.8, -0.13]

^*^The reference level: hospitalized but no oxygen therapy required

The primary ordinal outcome is the World Health Organization (WHO) 11-point clinical on day 14

The supplementary binary outcomes are as follows: (1) hospitalization at day 28, (2) the need for ventilation or worse at day 28, (3) mortality at day 28

## Discussion and conclusions

The current study presents a hierarchical framework for jointly modeling correlated mixed types of outcomes, which leads to improved precision in estimating heterogeneous treatment effects and optimal ITRs. The proposed Bayesian multivariate model leverages hierarchical modeling to effectively “borrow information” across outcomes, improving the estimation accuracy. Through extensive simulations, we compared the proposed model to a Bayesian univariate model, demonstrating that the proposed approach reduces the likelihood of making erroneous optimal ITRs. In the application to an international COVID-19 treatment trial, the proposed model exhibited better precision in estimating coefficients of treatment and treatment by pre-treatment characteristics interaction, as well as in estimating the OR for the primary ordinal outcome. This highlights the potential for improvement in clinical practice that the proposed model can offer through its applications in clinical research.

Our study should be interpreted considering two potential limitations. First, the framework is constrained to situations where the treatment effects and interaction effects are positively correlated and maintain a similar scale across outcomes. When these effects are negatively correlated with substantially different scales, our method would need to be adapted to account for such complex associations by introducing outcome-specific scales. Another potential approach is to use the ideas of group factor analysis [53, 54] to model both positive and negative relationships among outcomes by modeling the residuals as linear transformations of latent factors. To further explore the robustness of the proposed multivariate model, we conducted an additional simulation study to evaluate the impact of potential misspecifications of null effects as positively correlated effects, in which some effects were set to zero (e.g., the *A*-by-$\varvec{X}$ interaction effect coefficients and the treatment’s main effect coefficient are zero for one outcome) while others remained positively correlated. The simulation results indicate that although the inclusion of null effects resulted in somewhat reduced PCD and AUC compared to the case with only positively correlated outcome, the proposed multivariate approach still reduces the occurrence of erroneous treatment decisions in comparison to the univariate model, demonstrating that the proposed multivariate model remains relatively effective even when some effects are null. Detailed descriptions of this simulation study are provided in Additional File 8.

Second, the pre-treatment characteristics used for model fitting come from [24], representing the optimal variable set determined through cross-validation. We assessed the model’s goodness-of-fit using posterior predictive checking. The results show that the proposed multivariate model fits the data well, suggesting that the direct adoption of pre-treatment characteristics from [24] does not pose a serious limitation. When there is a definite expectation that specific pre-treatment characteristics will impact the outcome, those pre-treatment characteristics should be included in the model. When it is unclear whether certain pre-treatment characteristics should be included, data-dependent variable selection methods [55–59] can be incorporated.

We set the threshold $\delta$ to 0.5 to develop ITRs in Eq. (5) in this paper. However, there are clinical situations where different thresholds might be used based on the risk-benefit profile of the treatment. For treatments that carry significant risks or potential adverse effects, clinicians might prefer a higher threshold to ensure that only patients with a substantially higher probability of benefiting from a particular treatment are selected, decisively outweighing the risks.

In medical practice, therapy often consists of a series of treatments assigned in multiple stages, with clinicians choosing each treatment adaptively based on the patient’s treatment history and clinical outcomes at previous stages [60–63, 5, 64]. One potential avenue to expand the proposed approach is in the context of sequential treatment decisions [62, 5, 12, 65–75]. Developing effective methods for addressing the goal of optimizing individual treatment sequences is an important future research direction [60, 63, 65–67, 76]. We can envision that the secondary outcomes could be different at different stages of treatment. In addition, the primary outcome at one stage could have been a secondary outcome at a previous treatment stage. This line of investigations would lead to a complex setting, which would require further methodological developments. The methodological developments in sequential treatment decision making and precision medicine should match the complexity of human diseases and health care, and we expect that at first progress in this respect would be made by addressing a specific clinical situation, where clinical expertise would provide the outcomes’ ranking (importance) at different treatment stages.

To the best of our knowledge, no previous studies have jointly modeled mixed types of outcomes to develop ITRs. Our framework efficiently leverages information from multiple health outcomes, making it valuable for developing ITRs that utilize rich available outcome data.

## Supplementary Information

Additional file 1.

Additional file 2.

Additional file 3.

Additional file 4.

Additional file 5.

Additional file 6.

Additional file 7.

Additional file 8.

## Data Availability

The datasets used and analyzed during the current study are available from the corresponding author upon reasonable request.

## Abbreviations

ITRs: Individualized treatment decision rules
TBI: Treatment benefit index
COVID-19: Coronavirus disease 2019
COMPILE: COntinuous Monitoring of Pooled International Trials of ConvaLEscent Plasma for COVID-19 Hospitalized Patients
RCT(s): Randomized controlled trial(s)
CCP: COVID-19 convalescent plasma
WHO: World Health Organization
HMC: Hamiltonian Monte Carlo
$$\mathrm {OR(s)}$$: Odds ratio(s)
MVN: Multivariate normal distribution
AUC: Area under the curve
ROC: Receiver operating characteristic
PCD: Proportion of correct decisions
IQR: Interquartile range
SD: Standard deviation
CrI: Credible interval
MSE: Mean squared error
