# Supplementary material for: A Bayesian multivariate hierarchical model for developing a treatment benefit index using mixed types of outcomes
Source: BMC Med Res Methodol. 2024 Sep 27;24:218. doi: 10.1186/s12874-024-02333-z (PMC11437666; doi:10.1186/s12874-024-02333-z)
Supplement: Supplementary file 1 — Additional file 1. [file 12874_2024_2333_MOESM1_ESM.pdf]

Supplementary Materials

Additional file 1 — Stan code for the Bayesian multivariate hierarchical model

```
data {
  int<lower=0> N;                // number of observations
  int<lower=0> D;                // number of of binary outcomes
  int<lower=2> L;                // number of WHO categories
  int<lower=0> P_main;           // number of pre-treatment characteristics in the main effects term
  int<lower=0> P_inter;          // number of pre-treatment characteristics in the interaction effects term
  int<lower=1,upper=L> y_ord[N]; // vector of ordinal outcomes
  int<lower=0,upper=1> y_b[N,D]; // matrix of D binary outcomes (N x D matrix)
  int<lower=0,upper=1> A[N];     // treatment or control
  row_vector[P_main] x_main[N];  // pre-treatment characteristics in the main effects term  (N x P_main matrix)
  row_vector[P_inter] x_inter[N];// pre-treatment characteristics in the interaction effects term  (N x P_inter matrix)
}

parameters {
  ordered[L-1] tau;              // cut-points for cumulative odds model
  vector<lower=0>[(P_inter + 1)] sigma_beta; // sd of outcome-specific treatment main effect and interaction effect
  vector[D] beta_0;              // outcome-specific intercepts for D binary outcomes
  matrix[P_main,(D + 1)] beta_1; // covariates main effect for D binary outcomes and 1 ordinal outcome (P_main x (D + 1) matrix)
  vector[(P_inter + 1)] beta_star; // pooled treatment main effect and pooled interaction effect across all outcomes

  // non-central parameterization

  matrix[(P_inter + 1),(D + 1)] z_beta_int;
}

transformed parameters {
  matrix[(P_inter + 1),(D + 1)] beta_int; // outcome-specific treatment main effect and interaction effect ((P_inter + 1) x (D + 1) matrix)
  vector[D] yhat_b[N];
  real yhat_ord[N];

  for (j in 1:(P_inter + 1))
     for (k in 1:(D + 1)){
       beta_int[j,k] = beta_star[j] + sigma_beta[j] * z_beta_int[j,k];
  }

  for (i in 1:N){
    for (k in 1:D){
      yhat_b[i,k] =  beta_0[k] + x_main[i] * beta_1[,k]  +  (append_col(1, x_inter[i]) * beta_int[,k]) * A[i];
    }
    yhat_ord[i] = x_main[i] * beta_1[,D+1]  +  (append_col(1, x_inter[i]) * beta_int[,D+1]) * A[i];
  }
}


model {

  // priors

  sigma_beta ~ exponential(1);
  beta_star ~ normal(0,2.5);
```

```
  to_vector(beta_1) ~ normal(0,2.5);
  to_vector(z_beta_int) ~ std_normal();

  for (l in 1:(L-1)){
    tau[l] ~ student_t(3,0,8);
  }

   for (k in 1:D){
    beta_0[k] ~ student_t(3,0,8);
  }

  // outcome model

  for (i in 1:N){
    y_ord[i] ~ ordered_logistic(yhat_ord[i], tau);
    for (k in 1:D){
      y_b[i,k] ~ bernoulli_logit(yhat_b[i,k]);
    }
  }
}
```
