# Supplementary material for: A Bayesian multivariate hierarchical model for developing a treatment benefit index using mixed types of outcomes
Source: BMC Med Res Methodol. 2024 Sep 27;24:218. doi: 10.1186/s12874-024-02333-z (PMC11437666; doi:10.1186/s12874-024-02333-z)
Supplement: Supplementary file 2 — Additional file 2. [file 12874_2024_2333_MOESM2_ESM.pdf]

Additional file 2 — Comparison of multivariate and univariate models in terms of 95% credible interval length, coverage rate, and mean squared error

We conducted additional simulations to demonstrate that the proposed method reduces the uncertainty associated with the estimation of treatment effects. Using the simulation setup described in Section 2.3.1, we employed training sample sizes of $n \in \{250, 500\}$, and focused on the estimation of the treatment main effect for the primary ordinal outcome ($\beta_0^{(1)}$). For each sample size n, we conducted 1000 simulations and computed the following metrics:

1. The average length of the 95% credible intervals (CrIs) for $\beta_0^{(1)}$ across these simulations.
2. The coverage rate, defined as the percentage of simulations where the true value of the treatment effect ($\beta_0^{(1)}$) falls within the estimated 95% CrIs.
3. The mean squared error (MSE) between the estimated posterior median of the treatment effect and the true value.

The results, summarized in the Table A1, indicate that the proposed multivariate approach provides narrower credible intervals and lower MSE, while maintaining high coverage rate greater than 95%. This implies that the multivariate method is more desirable as it offers reduced uncertainty and more accurate estimations.

n	Model	95% credible interval length	Coverage rate	Mean squared error
250	Multivariate model (6)	1.11	96.35%	0.07
	Univariate model (7)	1.52	94.47%	0.16
500	Multivariate model (6)	0.78	97.25%	0.03
	Univariate model (7)	1.07	93.67%	0.08

Table A1 Comparison of multivariate and univariate models in terms of 95% credible interval length, coverage rate, and mean squared error
