# Supplementary material for: A Bayesian multivariate hierarchical model for developing a treatment benefit index using mixed types of outcomes
Source: BMC Med Res Methodol. 2024 Sep 27;24:218. doi: 10.1186/s12874-024-02333-z (PMC11437666; doi:10.1186/s12874-024-02333-z)
Supplement: Supplementary file 3 — Additional file 3. [file 12874_2024_2333_MOESM3_ESM.pdf]

Additional file 3 — Main analysis: comparing the performance of the Bayesian multivariate and univariate models when the true optimal ITR is determined by potential outcomes

To implement this potential outcomes-based ITR, we first consider the patient characteristics $\tilde{\boldsymbol{x}}_i$ along with the true values of parameters from the data generation process. Next, we use the *simstudy* package [44] to generate potential primary ordinal outcomes for subjects receiving the control treatment ($y^{(1)}_{A=0}$) and the experimental treatment ($y^{(1)}_{A=1}$). The optimal ITR is derived from the indicator function $I(y^{(1)}_{A=1} < y^{(1)}_{A=0})$, which evaluates whether the experimental treatment outcome is better than the control treatment outcome.

Utilizing this new potential outcomes-based ITR, the subsequent plot (Figure A1) illustrates the comparison of PCD and AUC values between the Bayesian multivariate and univariate models across varying training set sizes. In comparison to Figure 1,

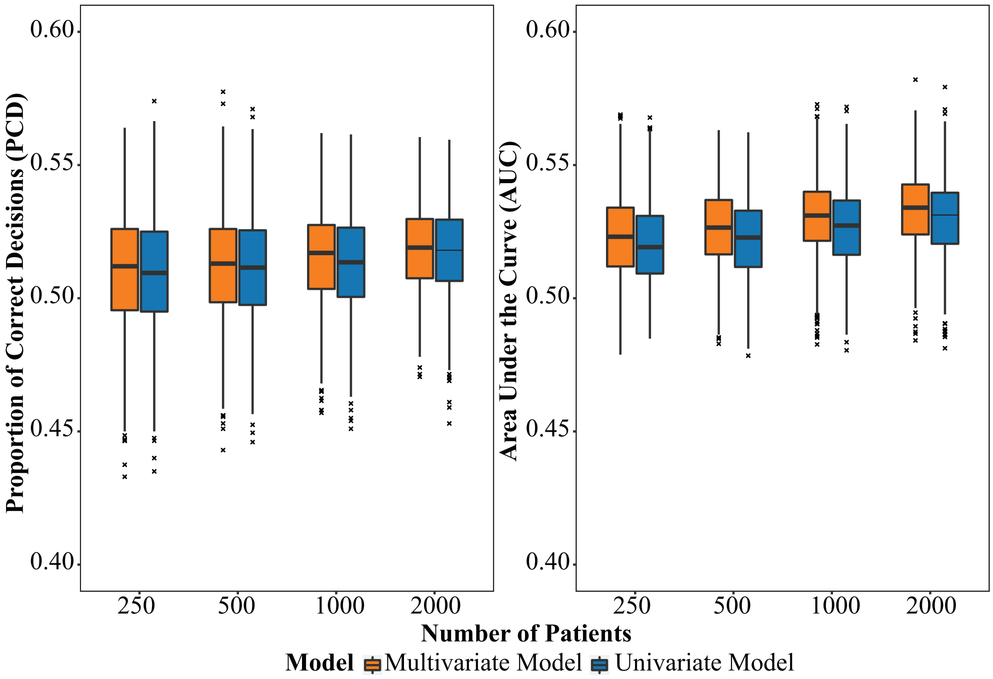

Fig. A1 Utilizing the new potential outcomes-based ITR: the boxplots of proportion of correct decisions (PCD) and area under the curve (AUC) in the test sets, comparing the multivariate (orange) and the univariate (blue) models across different training set sizes (as indicated in the x-axis).

the improvement in prediction using the multivariate model is less remarkable. This can be attributed to the fact that generating potential outcomes based on probability inherently involves more randomness. The gain in estimation is relatively small

compared to the magnitude of this randomness. Consequently, when considering prediction error, the improvement becomes less noticeable as it is overshadowed by the noise introduced by the randomness.
