# Supplementary material for: A Bayesian multivariate hierarchical model for developing a treatment benefit index using mixed types of outcomes
Source: BMC Med Res Methodol. 2024 Sep 27;24:218. doi: 10.1186/s12874-024-02333-z (PMC11437666; doi:10.1186/s12874-024-02333-z)
Supplement: Supplementary file 4 — Additional file 4. [file 12874_2024_2333_MOESM4_ESM.pdf]

Additional file 4 — Sensitivity analyses: comparing the performance of the Bayesian multivariate and univariate models when the true optimal ITR is determined by potential outcomes

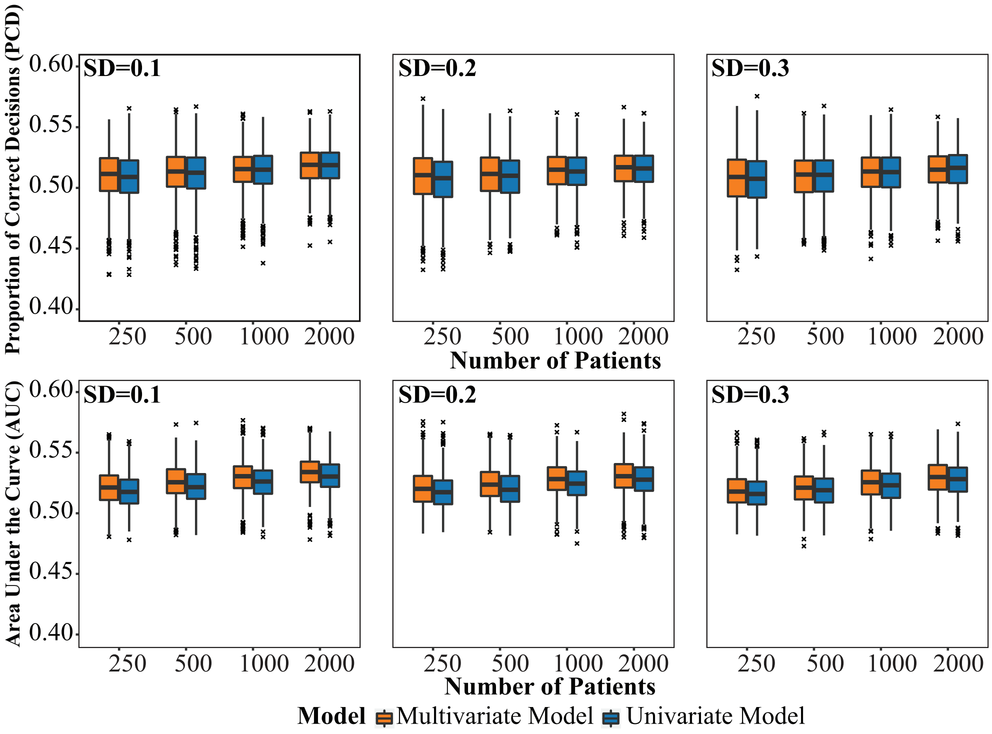

Fig. A2 Utilizing the potential outcomes-based ITR: the boxplots of proportion of correct decisions (PCD) and area under the curve (AUC) in the test sets, comparing the multivariate (orange) and univariate (blue) models across different training set sizes (as indicated in the x-axis) and different standard deviations (SDs) of random effects. Three different levels of SD for random effects are considered in data generation: SD=0.1, SD=0.2, and SD=0.3.

Compared to Figure 2, the utilization of this new potential outcomes-based ITR yields less remarkable improvement in the multivariate model's performance. This could be due to the probabilistic nature of generating potential outcomes, which inherently involves more randomness. Despite the gain in estimation, the magnitude of this randomness is relatively large, resulting in a small improvement that is overshadowed by the introduced noise when considering prediction error.
