# Supplementary material for: A Bayesian multivariate hierarchical model for developing a treatment benefit index using mixed types of outcomes
Source: BMC Med Res Methodol. 2024 Sep 27;24:218. doi: 10.1186/s12874-024-02333-z (PMC11437666; doi:10.1186/s12874-024-02333-z)
Supplement: Supplementary file 5 — Additional file 5. [file 12874_2024_2333_MOESM5_ESM.pdf]

Additional file 5 — Evaluating the robustness of the proposed multivariate model's results to different prior distributions.

We included additional sensitivity analyses to illustrate the robustness of our results to a different choice of prior.

We compared priors for σ_{β_j} (the standard deviation parameter) in the proposed multivariate model (6), using the simulation setup in Section 2.3.1 for training sample size $n \in \{250, 500\}$:

- $\sigma_{\beta_j} \sim exponential(\mu = 1)$ (Exponential distribution with mean 1)
- $\sigma_{\beta_j} \sim Cauchy(\mu = 0, \sigma = 1)$ (Cauchy distribution with location 0 and scale 1)

For each scenario, we re-ran the proposed multivariate Bayesian multivariate model with different priors, and compared them with respect to the PCD and AUC. The results of the sensitivity analysis are presented in Figure A3. We found that the exponential prior distribution leads to slightly higher PCD and AUC compared to the Cauchy prior distribution, but the difference is not dramatic or overwhelming, suggesting robustness of the results to this choice of priors.

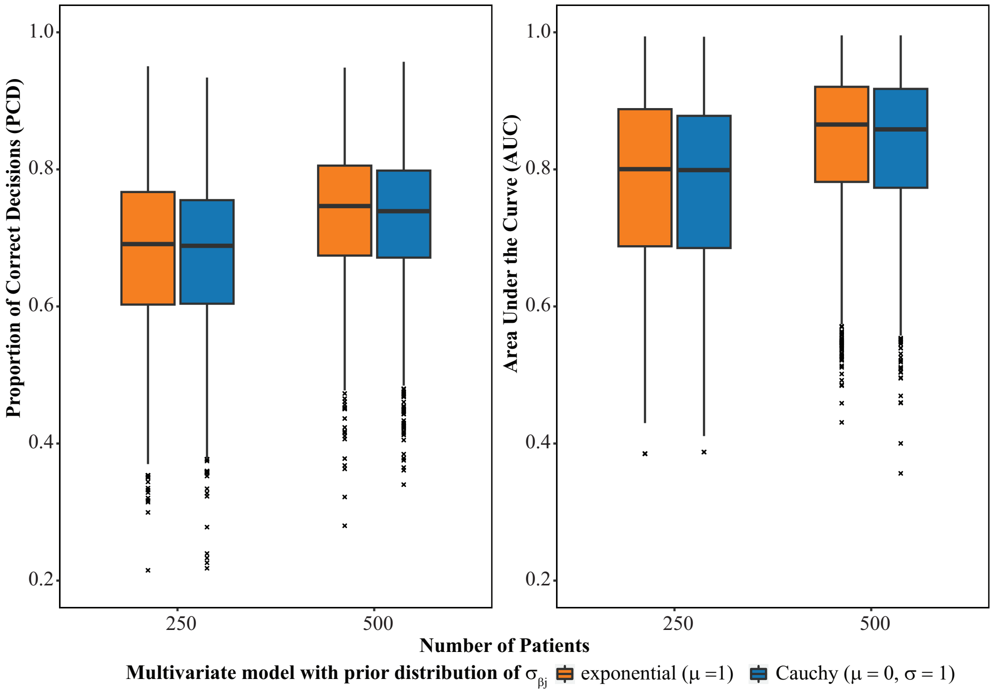

Fig. A3 Boxplots of the proportion of correct decisions (PCD) and area under the curve (AUC) in the test sets, comparing the multivariate model with prior distribution of $\sigma_{\beta_j} \sim$ exponential($\mu = 1$) (orange) and Cauchy($\mu = 0, \sigma = 1$) (blue) models across different training set sizes (as indicated in the x-axis). Each box shows the interquartile range (IQR), with the horizontal line inside the box representing the median PCD and AUC value. The whiskers extend to the minimum and maximum PCD and AUC values within 1.5 times the IQR. Outliers are represented by small cross symbols.
