# Supplementary material for: A Bayesian multivariate hierarchical model for developing a treatment benefit index using mixed types of outcomes
Source: BMC Med Res Methodol. 2024 Sep 27;24:218. doi: 10.1186/s12874-024-02333-z (PMC11437666; doi:10.1186/s12874-024-02333-z)
Supplement: Supplementary file 6 — Additional file 6. [file 12874_2024_2333_MOESM6_ESM.pdf]

Additional file 6 — The World Health Organization (WHO) 11-point COVID-19 clinical status scale.

0:	Uninfected, no viral RNA detected
1:	Asymptomatic, viral RNA detected
2:	Symptomatic, independent
3:	Symptomatic, assistance needed
4:	Hospitalized, no oxygen therapy
5:	Hospitalized, oxygen by mask or nasal prongs
6:	Hospitalized, oxygen by non-invasive ventilation or high flow
7:	Intubation & mechanical ventilation, $pO_2/FiO_2 \geq 150$ (or $SpO_2/FiO_2 \geq 200$)[a]
8:	Mechanical ventilation, $pO_2/FiO_2 < 150$ (or $SpO_2/FiO_2 < 200$) or vasopressors
9:	Mechanical ventilation, $pO_2/FiO_2 < 150$ and vasopressors, dialysis, or ECMO[b]
10:	Dead

[a] pO_2: partial pressure of oxygen, FiO_2: fraction of inspired oxygen, SpO_2: oxygen saturation.
[b] ECMO: extracorporeal membrane oxygenation.

Table A2 The World Health Organization (WHO) 11-point COVID-19 scale definition[41].

	**Control (n = 1097)**	**CCP (n = 1190)**
WHO = 0	114	150
WHO = 1	151	168
WHO = 2	365	386
WHO = 3	134	142
WHO = 4	45	45
WHO = 5	86	84
WHO = 6	29	52
WHO = 7	23	30
WHO = 8	23	36
WHO = 9	33	22
WHO = 10	94	75

[a] CCP: COVID-19 convalescent plasma.
[b] WHO: The World Health Organization 11-point COVID-19 scale.

Table A3 The number of patients at different clinical stages of COVID-19 measured on the WHO 11-point scale at day 14 by treatment group.
