# Supplementary material for: A Bayesian multivariate hierarchical model for developing a treatment benefit index using mixed types of outcomes
Source: BMC Med Res Methodol. 2024 Sep 27;24:218. doi: 10.1186/s12874-024-02333-z (PMC11437666; doi:10.1186/s12874-024-02333-z)
Supplement: Supplementary file 7 — Additional file 7. [file 12874_2024_2333_MOESM7_ESM.pdf]

Additional file 7 — Goodness-of-fit using posterior predictive checking

In evaluating the suitability of a statistical model, it is crucial to determine whether the model provides an accurate representation of the observed data. This is particularly important for models like the cumulative proportional odds model, which is built upon a strong assumption of proportional cumulative odds. Posterior predictive checking serves as an effective way of assessing a model's *goodness-of-fit* [48, 49]. This method operates on the premise that a well-fitted model should enable the generation of replicated data (D^{rep}) that resembles the observed data (D^{original}) [50].

The lack of fit can be measured by the Bayesian p-value, which represents the probability of the test statistic (e.g., $P(Y \leq y), y = 0, \ldots, 9$) for D^{rep} being equal to or exceeding the test statistic for D^{original}. A Bayesian p-value approaching zero or one signifies a potential issue with the model's fit, whereas a value near 0.5 suggests that the model captures the data well [43, 79]. We employed the procedure outlined in [40] to examine the cumulative proportional odds model's fit to the observed data and to compute the Bayesian p-value.

Table A4 provides the results of posterior predictive checking based on ten test statistics (along with their 95% CrIs) for both the multivariate model (6) and univariate model (7). Our analysis confirmed the satisfactory fit of both models to the data.

Treatment	**Control**			**CCP**		
Test quantity: % subjects	$T(D^{\text{original}})$	95% int. for $T(D^{\text{rep}})$	Bayesian P value	$T(D^{\text{original}})$	95% int. for $T(D^{\text{rep}})$	Bayesian P value
**Multivariate model**						
WHO $\leq$ 0	10.39	[8.39, 12.94]	0.55	12.61	[10.08, 14.71]	0.39
WHO $\leq$ 1	24.16	[20.51, 26.80]	0.34	26.72	[23.28, 29.58]	0.41
WHO $\leq$ 2	57.43	[52.42, 59.43]	0.19	59.16	[55.71, 62.52]	0.48
WHO $\leq$ 3	69.64	[65.36, 71.93]	0.29	71.09	[68.24, 74.45]	0.55
WHO $\leq$ 4	73.75	[69.92, 76.12]	0.32	74.87	[72.44, 78.32]	0.63
WHO $\leq$ 5	81.59	[78.21, 83.68]	0.34	81.93	[80.17, 85.29]	0.74
WHO $\leq$ 6	84.23	[82.22, 87.15]	0.64	86.30	[83.78, 88.40]	0.44
WHO $\leq$ 7	86.33	[84.78, 89.43]	0.74	88.82	[86.13, 90.50]	0.33
WHO $\leq$ 8	88.42	[87.69, 91.89]	0.90	91.85	[88.82, 92.69]	0.14
WHO $\leq$ 9	91.43	[90.43, 94.07]	0.81	93.70	[91.26, 94.71]	0.22
**Univariate model**						
WHO $\leq$ 0	10.39	[8.57, 13.13]	0.62	12.61	[9.83, 14.54]	0.32
WHO $\leq$ 1	24.16	[20.78, 27.07]	0.41	26.72	[23.03, 29.41]	0.36
WHO $\leq$ 2	57.43	[52.42, 59.80]	0.23	59.16	[55.55, 62.44]	0.46
WHO $\leq$ 3	69.64	[65.45, 72.20]	0.31	71.09	[68.15, 74.45]	0.55
WHO $\leq$ 4	73.75	[69.83, 76.30]	0.34	74.87	[72.44, 78.40]	0.62
WHO $\leq$ 5	81.59	[78.12, 83.87]	0.35	81.93	[80.17, 85.29]	0.74
WHO $\leq$ 6	84.23	[82.04, 87.24]	0.64	86.30	[83.78, 88.49]	0.45
WHO $\leq$ 7	86.33	[84.69, 89.52]	0.74	88.82	[86.13, 90.50]	0.34
WHO $\leq$ 8	88.42	[87.60, 91.89]	0.89	91.85	[88.82, 92.69]	0.14
WHO $\leq$ 9	91.43	[90.34, 94.17]	0.81	93.70	[91.34, 94.79]	0.23

[a]WHO: The World Health Organization 11-point COVID-19 scale.

Table A4 Summary of posterior predictive checking based on the ten test statistics.
