# Supplementary material for: A Bayesian multivariate hierarchical model for developing a treatment benefit index using mixed types of outcomes
Source: BMC Med Res Methodol. 2024 Sep 27;24:218. doi: 10.1186/s12874-024-02333-z (PMC11437666; doi:10.1186/s12874-024-02333-z)
Supplement: Supplementary file 8 — Additional file 8. [file 12874_2024_2333_MOESM8_ESM.pdf]

Additional file 8 — Evaluating the multivariate model performance on scenarios with a mix of null and positively correlated effects across outcomes

In this simulation, we followed the same setup as described in Section 2.3.1, but with an additional outcome where the treatment-by-covariates (A-by-$\boldsymbol{X}$) interaction effect coefficients and the treatment's main effect coefficient are set to 0 for this outcome:

For a given training sample size $n \in \{250, 500\}$, we independently generated treatment indicators, denoted $A_i \in \{0, 1\}$, from the Bernoulli distribution with a probability of $P(A_i = 1) = 0.5$. The covariates $\boldsymbol{X}_i \in \mathbb{R}^p$ comprised 3 independent binary variables generated from the Bernoulli distribution with probability $P(X_i = 1) = 0.5$, and $p - 3$ independent continuous variables, drawn from the multivariate normal distribution with mean zero and unit variance. We considered $p = 5$ covariates.

We generated a set of five outcomes $(Y_i^{(1)}, Y_i^{(2)}, Y_i^{(3)}, Y_i^{(4)}, Y_i^{(5)})$, where the primary outcome $Y_i^{(1)}$ followed an 11-level ordinal multinomial distribution, while the other 4 outcomes ($Y_i^{(2)}$, $Y_i^{(3)}$, $Y_i^{(4)}$ and $Y_i^{(5)}$) correspond to "supplementary" binary outcomes, with the 5th outcome $Y_i^{(5)}$ not associated with the treatment.

The true parameter values used for the data generation are as follows. The covariates' main effect coefficients for each of the 5 outcomes are: $\boldsymbol{m}^{(1)} = [0.35, -0.40, 0.15, 0.20, -0.21]^\top$, $\boldsymbol{m}^{(2)} = [0.40, -0.38, 0.13, 0.19, -0.22]^\top$, $\boldsymbol{m}^{(3)} = [0.38, -0.39, 0.14, 0.18, -0.20]^\top$, $\boldsymbol{m}^{(4)} = [0.42, -0.41, 0.16, 0.21, -0.19]^\top$, $\boldsymbol{m}^{(5)} = [0.41, -0.40, 0.15, 0.20, -0.20]^\top$.

- Treatment's main effect coefficient for each outcome:
  - $\beta_0^{(1)} = -0.05$
  - $\beta_0^{(2)} = -0.06$
  - $\beta_0^{(3)} = -0.03$
  - $\beta_0^{(4)} = -0.04$
  - $\beta_0^{(5)} = 0$
- A-by-$\boldsymbol{X}$ interaction effect coefficients for each outcome:
  - $\boldsymbol{\beta}^{(1)} = \begin{bmatrix}0.20 & -0.10 & 0.10 & 0.05 & -0.06\end{bmatrix}^\top$
  - $\boldsymbol{\beta}^{(2)} = \begin{bmatrix}0.19 & -0.11 & 0.09 & 0.04 & -0.07\end{bmatrix}^\top$
  - $\boldsymbol{\beta}^{(3)} = \begin{bmatrix}0.18 & -0.12 & 0.11 & 0.06 & -0.05\end{bmatrix}^\top$
  - $\boldsymbol{\beta}^{(4)} = \begin{bmatrix}0.21 & -0.09 & 0.12 & 0.07 & -0.04\end{bmatrix}^\top$
  - $\boldsymbol{\beta}^{(5)} = \begin{bmatrix}0 & 0 & 0 & 0 & 0\end{bmatrix}^\top$

We compared the performance of the proposed multivariate model (6) that uses these 5 outcomes with that using only the 4 positively correlated outcomes $\{Y_i^{(1)}$, $Y_i^{(2)}$, $Y_i^{(3)}$, $Y_i^{(4)}\}$, and with the univariate model (7) that only uses the single primary ordinal outcome.

The results of this additional simulation study are presented in Figure A4. We found that, although the inclusion of null effects resulted in somewhat reduced PCD

and AUC, the multivariate model still reduces the occurrence of erroneous treatment decisions compared to the univariate model for a single outcome, demonstrating that the proposed multivariate model can reasonably handle scenarios with a mix of null and positively correlated effects.

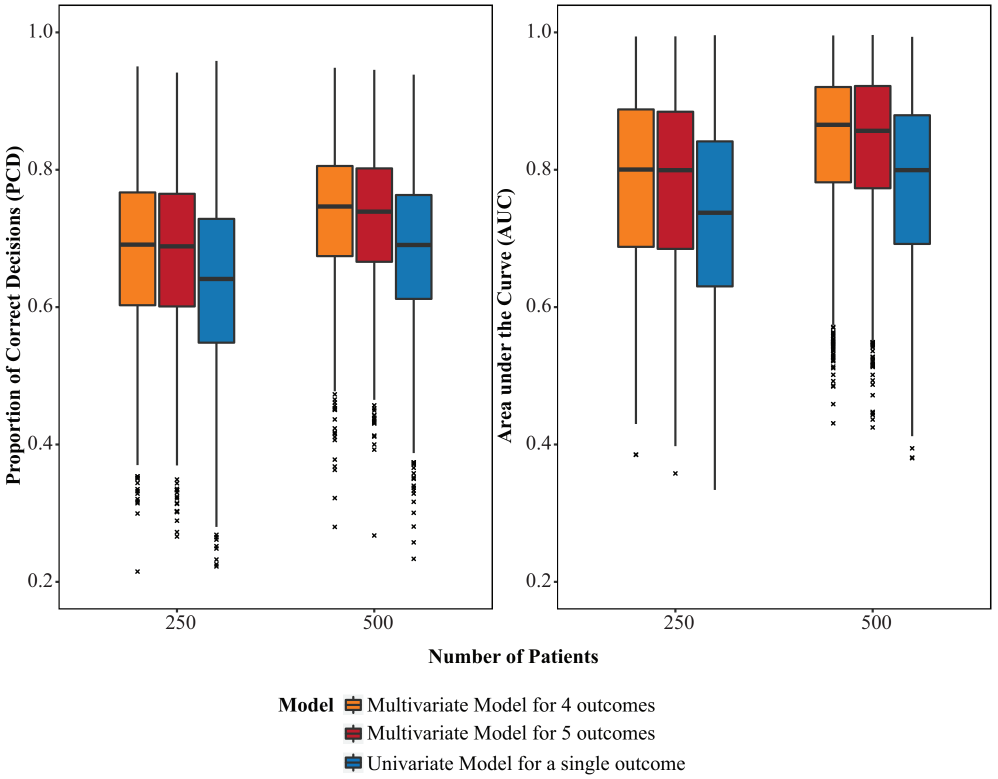

Fig. A4 Boxplots of the proportion of correct decisions (PCD) and the area under the curve (AUC) in the test sets, comparing different models, when some effects are null and others are positively correlated. The results are from three approaches: the multivariate model using 4 positively correlated outcomes (orange), the multivariate model using 5 outcomes including null effects (red), and univariate model using only a single primary outcome (blue), across different training set sizes (as indicated on the x-axis). Each box shows the interquartile range (IQR), with the horizontal line inside the box representing the median PCD and AUC value. The whiskers extend to the minimum and maximum PCD and AUC values within 1.5 times the IQR. Outliers are represented by small cross symbols.
